# Genetic variation of the 5‐HT1A rs6295, 5‐HT2A rs6311, and CNR1 rs1049353 and an altered endocannabinoid system in depressed patients

**DOI:** 10.1002/brb3.3323

**Published:** 2023-11-20

**Authors:** Jasmin Obermanns, Hanna Meiser, Saskia Hoberg, Cynthia Segura Vesterager, Frank Schulz, Georg Juckel, Barbara Emons

**Affiliations:** ^1^ LWL University Hospital Department of Psychiatry Psychotherapy and Preventive Medicine Ruhr University Bochum Bochum Germany; ^2^ Chemistry and Biochemistry of Natural Products Ruhr University Bochum Bochum Germany

**Keywords:** 2‐arachidonoylglycerol, arachidonoyl ethanolamide, cannabinoid receptor, depression, serotonin receptor

## Abstract

**Background:**

The reasons for developing depression are not fully understood. However, it is known that the serotonergic system plays a role in the etiology, but the endocannabinoid system receives attention.

**Method:**

In this study, 161 patients with a depressive disorder and 161 healthy participants were examined for the distribution of the CNR1 rs4940353, 5‐HT2A rs6311, and 5‐HT1A rs6295 by high‐resolution melting genotyping. The concentration of arachidonoyl ethanolamide (AEA) and 2‐arachidonoylglycerol (2‐AG) in the blood was measured by liquid chromatography–tandem mass spectrometry. Additionally, depression and anxiety symptoms were evaluated based on self‐questionnaires. Fifty‐nine patients participated in a second appointment to measure the concentration of AEA, 2‐AG, and symptoms of depression and anxiety.

**Results:**

We observed higher AEA and decreased 2‐AG concentrations in patients with depression compared to healthy participants. During the treatment, the concentrations of AEA and 2‐AG did not change significantly. In patients higher symptoms of anxiety correlated with lower concentrations of 2‐AG. Gender differences were found concerning increased 2‐AG concentration in male patients and increased anxiety symptoms in female patients. Genotypic variations of 5‐HT1A rs6295 and 5‐HT2A rs6311 are associated with altered serotonergic activity and serotonin content in patients.

**Conclusion:**

In conclusion, it seems that the endocannabinoid system, especially the endocannabinoids 2‐AG and AEA, and genetic variations of the 5‐HT1A and 5‐HT2A could play a role in patients with depression and may be involved in a depressive disorder.

## Abstract 2

The present study showed an altered endocannabinoid system and the genetic variations of the 5‐HT1A, 5‐HT2A, and CNR1 genes in patients with a depressive disorder compared to healthy participants.

## 1 SIGNIFICANCE

The origin of depressive disorders has not yet been fully elucidated. Nevertheless, it is now known that the cause of depression is multifunctional and is based on complexity. Therefore, it is even more important to identify and investigate these factors and possible interactions. This study aimed to examine potential interactions of the serotonergic system with the endocannabinoid system in depressed patients and how far genetic predispositions may influence the disease. The results describe an altered endocannabinoid system and an influence of genetic predispositions in the serotonergic system in patients with a depressive disorder. Furthermore, the results underline the complexity of this disorder.

## 2 INTRODUCTION

The serotonin system and its receptors play a crucial role in the central nervous system processes modulating mood, stress, sleep, and emotion. Recent studies have shown a relationship between the genes encoding the serotonin 1A and 2A receptors (5‐HT1A and 5‐HT2A) receptor, and depressive disorders (Celada et al., 2004; Kishi et al., 2009). The 5‐HT1A receptor has a high density in limbic and cortical regions in the brain. The receptors function as presynaptic somatodendritic autoreceptors and postsynaptic heteroreceptors. As a presynaptic autoreceptor, it mediates a negative feedback inhibition on 5‐HT neurons (Kaufman et al., 2016). One of the most investigated gene variants on the serotonin receptor 1A is the 5‐HT1A C(−1019)G (rs6295) polymorphism. This functional polymorphism is located in the promoter region of the *Htr1A* gene. In numerous studies, the rs6295 polymorphism could be associated with depression and anxiety disorders (Albert, 2012; Domschke et al., 2006; Fakra et al., 2009; Parsey et al., 2010). Especially, the G‐allele of the rs6295 is associated with depression (Kim et al., 2011; Molina et al., 2011; Parsey et al., 2006).

5‐HT2A is a postsynaptic autoreceptor located in the frontal cortex, which applies to the motor system and plays a primary role in the limbic system in the brain (Köhler et al., 2016). One of the most investigated polymorphisms of the 5‐HT2A receptor is rs6311. With regard to the polymorphism in the 5‐HT2A receptor (rs6311), studies suggest that it is a susceptibility factor for the development of depression and plays a role in antidepressant therapy (Busch & Menke, 2019; Cao et al., 2015; Pandey et al., 2010).

The results of the influence of 5‐HT1A and 5‐HT2A receptor genes are inconsistent (Tencomnao et al., 2010). It is essential to clarify the role of these genetic variations in patients with a depressive disorder and their relevance in the etiology of depression.

In addition to the serotonergic system, a possible role of ECS in the pathophysiology of depressive disorders has already been described. Previous studies suggest that depressive disorders may be associated with deficient endocannabinoid signaling (Rana et al., 2021). In patients with depressive disorder, concentrations of arachidonoyl ethanolamide (AEA) and 2‐arachidonoylglycerol (2‐AG) were decreased, with 2‐AG concentration correlating negatively with the duration of the depressive episode (Hill et al., 2008, 2009). Further studies postulated that concentrations of AEA and 2‐AG were increased after treatment with selective serotonin reuptake inhibitors compared with healthy controls (Romero‐Sanchiz et al., 2019). Evidence from postmortem studies described increased CB1 availability in depression (Choi et al., 2012; Mato et al., 2018). Several studies have reported associations between genetic variations in the CB1 receptor (CNR1) and susceptibility to developing depression (see review Bright & Akirav, 2022). Patients who developed a depressive disorder showed higher frequencies of the mutated gene in the CNR1 gene (Juhasz et al., 2009; Monteleone et al., 2010).

It is already clear that depressive disorders are the result of interactions between a variety of systems and that the causes are multifactorial. A possible interaction of the serotonergic system with the endocannabinoid system cannot be excluded, as cannabinoid signaling has been implicated in the regulation of some serotonergic functions (Häring et al., 2015) and cannabinoids can modulate serotonin 2A (5‐HT2A) receptor signaling in the brain (Ibarra‐Lecue, Mollinedo‐Gajate, et al., 2018; Ibarra‐Lecue, Pilar‐Cuéllar, et al., 2018).

In this study, we examined the association among 5‐HT1A (rs6295), 5‐HT2A (rs6311), and CNR1 (rs1049353) and measured the concentration of AEA and 2‐AG in patients with depression compared to healthy controls. In a previous study, we had already investigated the relationship between serotonergic activity and the concentration of serotonin (serum/platelet) in patients with depression (Obermanns et al., 2022). Moreover, we hope to find a link between the serotonergic system and the endocannabinoid system in the pathology of depression.

## 3 MATERIALS AND METHODS

### 3.1 Subjects

The subjects in this study were 161 patients and 161 age‐ and sex‐matched healthy participants (100 females and 61 males, average age 39 ± 11). All individuals had given their written informed consent. The patients were diagnosed according to the International Statistical Classification of Diseases and Related Health Problems (ICD‐10). Patients and healthy participants who abused drugs or alcohol and exhibited neurological and serious internal diseases were excluded. The healthy participants did not have any psychiatric disorders. The patients were recruited at the LWL University Hospital for Psychiatry, Psychosomatic and Preventive Medicine in Bochum, Germany. The study was accepted by the Ethics Committee of the Medical Faculty of the Ruhr University Bochum (ethics application number 5121‐14).

### 3.2 Study design

The present study included patients with depressive disorder and healthy participants. The study cohort was separated into subgroups in which different parameters were examined. First, all patients (*n* = 161) and healthy controls (*n* = 161) included in the study underwent fasting blood withdrawal, and the genetic variability of polymorphisms in the genes CNR1 rs1049353, 5‐HT1A rs6295, 5‐HT2A rs6311 was investigated. Furthermore, the patients and the healthy participants completed psychometric questionnaires.

In the first subgroup, besides genotyping, the endocannabinoid concentration of AEA and 2‐AG was determined at T1 and T2 (28 days later and 8–10 weeks later, respectively) in patients and healthy controls (T1). One part of the participant samples was examined in a screening laboratory in Hannover (*N* = 144 (T1)/*n* = 59 (T2)). The other part of the sample was collected at a later time and measured at the Ruhr University Bochum (*N* = 178 (T1)/*n* = 31 (T2)). At the second study visit, the patients completed the self‐rating questionnaires again.

To investigate a possible relation between the serotonergic and endocannabinoid system, besides genotyping the serotonin concentration in serum and platelets, as well as the serotonergic activity (loudness dependence of auditory evoked potentials [LDAEP]) in the brain at T1, was measured in the second subgroup consisting of patients with a depressive disorder (*n* = 89) and healthy participants (*n* = 89).

### 3.3 Psychometric instruments

All patients and healthy participants completed the following psychometric questionnaire Hamilton Depression Scale (HAMD), Beck Depression Inventory (BDI), and the State‐Trait Anxiety Inventory (STAI) and participated in a neuropsychiatric interview (M.I.N.I) for classification purposes (Ackenheil et al., 1999). The severity of depression was assessed by the HAMD (Hamilton, 1960). Likewise, the BDI (Hautzinger et al., 2006) and STAI (Laux et al., 1981) were used as self‐rating questionnaires.

### 3.4 Measurement of 2‐AG and anandamide

The concentrations of the endocannabinoids AEA and 2‐AG were measured in collaboration with the Screening Laboratory Hannover by mass spectrometry. Measurements were performed by ultra‐performance liquid chromatography combined with tandem mass spectrometry (Zoerner et al., 2012).

We determined plasma concentrations of AEA and 2‐AG simultaneously. Sampling and processing were performed based on a blood withdrawal into ethylenediaminetetraacetic acid (EDTA) tubes (Sarstedt) followed by centrifugation (3500 rpm, 4°C). The samples were frozen in a freezer (−80°C) until they were analyzed. The stored samples were thawed on ice. To reach a final concentration of 2.5 nM, 300‐μL aliquots were spiked with a mixture of deuterated (d) analogs in ethanol (d4‐AEA and d5‐2AG). The d4‐AEA and d5‐2AG served as internal standards for AEA and 2‐AG. Plasma samples were incubated on ice for 15 min. The extraction was performed by adding 1 mL of toluene to each sample and shaking twice in a homogenizer at 5000 rpm for 20 s, with an interruption of 5 s so that the heating of the samples was avoided. Phase separation was followed by centrifugation (14,500 rpm, 4°C, 5 min). The upper organic phase was transferred to a 1.5‐mL glass vial. After evaporating the solvent under a nitrogen stream, the residue was dried at room temperature (25°C) under nitrogen. Residues were reconstituted in 40 µL water–methanol (1:3, v/v), mixed by vortexing for 10 s, and 10‐µL aliquots were injected. The quantitative analyses were carried out with positive electrospray ionization and selected reaction monitoring as described by Zoerner et al. (2012).

This study included another group of subjects where the time between the first and second study visits was 8–10 weeks. Despite extensive method variations in terms of mobile phase, columns, and extraction methodology, beginning with the previously established procedures (Zoerner et al., 2012), no endocannabinoids could be detected in this sample group. A degradation of endocannabinoids during sample collection, processing, storage, or transport, with residual quantities being potentially adsorbed by polypropylene‐based storage tubes, cannot be excluded (Kratz et al., 2021; Rouzer et al., 2002).

### 3.5 Serotonin serum measurements

For the serotonin measurements, all participants take part in fasting blood withdrawal through venipuncture into EDTA tubes and serum tubes (Sarstedt). Serum tubes were kept frozen at −80°C until assayed. Serum serotonin content was measured using the Serotonin ELISA kit from Enzo Life Science and according to the manufacturer's instructions. The serum serotonin content was calculated by using a standard straight line.

### 3.6 Measurements of the serotonergic activity in the brain (loudness dependence of auditory evoked potentials [cortical LDAEP]; LORETA [source LDAEP])

The serotonergic activity in the brain was measured with the noninvasive LDAEP method. The performance and instructions of the LDAEP (cortical and source) can be taken from Obermanns et al. (2022).

### 3.7 Genotyping of the single nucleotide polymorphism (SNP) of the 5‐HT2A (rs6311‐1438 A/G), 5‐HT1A (rs6295‐1019 C/G), CNR1 (rs1049353)

The genomic DNA of patients and healthy participants was extracted from EDTA tubes (Sarstedt) using the Qiagen Blood Mini Kit (Qiagen) according to the manufacturer's instructions. The DNA samples were subsequently diluted to a concentration of 10 ng/µL.

The rs6295 (5‐HT1A), rs6311 (5‐HT2A), and rs1049353 (CNR1) were analyzed using high‐resolution melting (HRM) genotyping. HRM analysis uses different denaturation characteristics of the nucleotides to determine the DNA sequence. Before HRM analysis with the Precision Melt Analysis Software (BioRad), the DNA sequence of interest was amplified in a qPCR. The qPCRs were carried out on a CFX384 Touch Real‐Time PCR Detection System (BioRad).

The qPCR reaction for the genotyping contained 20 ng of DNA, 0.2 µM primers (5‐HT1A fw: 5′‐TTG ATG GAA GAA GAC CGA GTG‐3′, rev: 5′‐TTG ATG GAA GAA GAC CGA GTG‐3′; 5‐HT2A fw: 5′‐GTT GGC TTT GGA TGG AAG TGC‐3′, rev: 5′‐GTA TGT CCT CGG AGT GCT GT‐3′; CNR1 fw: 5′‐GTT CAC AGG GCC GCA GAA A‐3′, rev: 5′‐ GTG GAC ACA GAC ATG GTT ACC T‐3′) and 10 μL precision melt supermix for HRM analysis (BioRad) in a total volume of 20 µL. The amplification protocol included an initial denaturation step for 3 min at 95°C, followed by 40 cycles of melting at 95°C for 5 s, annealing and extension at 60°C for 30 s, and a melting curve analysis. All experiments were performed in duplicates and included non‐template controls.

### 3.8 Statistical analysis

To determine the correlations within patients with depression and healthy participants, Pearson's correlation was used. For the correlation, only the matched pairs where all values were present were included (*n* = 72).

The distribution and presence of Hardy Weinberg equilibrium were tested with the chi‐squared test (*χ* ^2^‐test) for best fit. The *χ* ^2^‐test was used to determine the statistical significance of the difference between the genotype frequencies. Differences between the groups were tested with an independent two‐sample *t*‐test. To calculate the distinction in the patients’ group (baseline/follow‐up), the dependent two‐sample *t*‐test was used. ANOVA was used to investigate the differences between the genotypes (5‐HT1A, 5‐HT2A, CNR1) and endocannabinoids (AEA, 2‐AG), psychometry (HAMD, BDI, STAI), serotonin content (serum, platelet), and serotonergic activity (LDAEP). Statistical analysis was conducted using IBM SPSS Statistic Software (IBM Corp., Version 25.0.).

## 4 RESULTS

### 4.1 Questionnaires

There were significant differences between the group of patients and healthy participants. Compared to the healthy participants, patients have more severe symptoms of depression and anxiety (Table 1).

**TABLE 1:** Demographics, clinical data, endocannabinoid concentration, and psychometry data of patients and healthy participants.

	Patients	Healthy participants
Number of subjects	161	161
Age	39.34 ± 11.67	39.25 ± 11.90
Diagnosis		
F31.X (*n*/%)	11 (6.8%)	
F32.X (*n*/%)	70 (43.5%)	
F33.X (*n*/%)	80 (49.7%)	
Gender (*n*/%)		
Female	100 (62.1%)	100 (62.1%)
Male	61 (37.9%)	61 (37.9%)
Psychometry		
HAMD‐21 (*n* = 161) (M ± SD)	20.01 ± 5.60	1.16 ± 1.69
*t*‐Test	*t*(188.82) = 40.93, *p* = .000***	
BDI‐II (*n* = 161) (M ± SD)	33.27 ± 9.93	2.93 ± 4.30
*t*‐Test	*t*(217.99) = 35.57, *p* = .000***	
STAI‐X1 (*n* = 161) (M ± SD)	57.22 ± 12.09	32.12 ± 7.36
*t*‐Test	*t*(264.26) = 22.50, *p* = .000***	
STAI‐X2 (*n* = 144) (M ± SD)	60.89 ± 9.55	32.04 ± 7.04
*t*‐Test	*t*(263.06) = 29.18, *p* = .000***	
Endocannabinoids		
AEA (nM) (*n* = 72) (M ± SD)	1.04 ± 0.75	0.80 ± 0.40
*t*‐Test	*t*(108.64) = 2.46, *p* = .015**	
2‐AG (nM) (*n* = 69) (M ± SD)	2.91 ± 1.42	4.14 ± 2.27
*t*‐Test	*t*(114.205) = −3.789, *p* = .000***	

Abbreviation: 2‐AG, 2‐arachidonoylglycerol; AEA, arachidonoyl ethanolamide; BDI‐II, Beck depression inventory; HAMD‐21, Hamilton; M, mean value; SD, standard deviation; STAI‐X1, state anxiety inventory; STAI‐X2, trait anxiety inventory.

**p < .01, ***p < .001.

After 4 weeks, we found higher scores in the HAMD, and BDI at T1 compared to T2, which indicates an improvement in depressive symptoms (Table 2).

**TABLE 2:** Endocannabinoid concentration and psychometry of patients during the treatment (T1/T2).

	Patients (T1)	Patients (T2)
Number of subjects	59	
Age	41 ± 10	
Endocannabinoids		
AEA (nM) (*n* = 59) (M ± SD)	1.09 ± 0.82	0.099 ± 0.69
*t*‐Test	*t*(58) = 1.027, *p* = .309	
2‐AG (nM) (*n* = 56) (M ± SD)	2.72 ± 1.31	2.72 ± 1.65
*t*‐Test	*t*(55) = .018, *p* = .986	
Psychometry		
HAMD‐21 (*n* = 59) (M ± SD)	27.71 ± 5.86	16.97 ± 17.04
*t*‐Test	*t*(58) = 2.207, *p* = .031*	
BDI‐II (*n* = 59) (M ± SD)	33.86 ± 9.53	28.34 ± 18.51
*t*‐Test	*t*(58) = 2.295, *p* = .025*	
STAI‐X1 (*n* = 59) (M ± SD)	58.56 ± 10.35	55.07 ± 16.33
*t*‐Test	*t*(58) = 1.603, *p* = .114	
STAI‐X2 (*n* = 59) (M ± SD)	59.32 ± 10.74	57.17 ± 15.64
*t*‐Test	*t*(58) = 1.027, *p* = .309	

Abbreviations: 2‐AG, 2‐arachidonoylglycerol; AEA, arachidonoyl ethanolamide; BDI‐II, Beck depression inventory; HAMD‐21, Hamilton; M, mean value; SD, standard deviation; STAI‐X1, state anxiety inventory; STAI‐X2, trait anxiety inventory.

*p < .05.

Moreover, the scores of the STAI‐X1 and STAI‐X2, which reflect trait anxiety, were not significantly reduced at T2 (Table 2). With regard to gender differences, females reached significantly higher scores in the STAI‐X1 than males (female (*n* = 100) = 58.84 ± 11.89; male (*n* = 61) = 54.57 ± 12.05; *t*(159) = 2.20; *p* = .029). Female patients with a depressive disorder have more anxiety symptoms than male patients.

### 4.2 Measurements of the endocannabinoids AEA and 2‐AG in patients (T1/T2) and healthy participants

The endocannabinoid content of AEA and 2‐AG in patients with a depressive disorder and healthy participants showed significant differences. The concentration of 2‐AG was significantly reduced in patients compared to healthy participants (*t*(114.205) = −3.789, *p* = .000) (Table 1). In contrast, the concentration of AEA was raised in patients compared to the healthy group (*t*(108.636) = 2.462, *p* = .015) (Table 1).

The concentration of endocannabinoids AEA and 2AG did not change significantly during the treatment. The follow‐up value of AEA is slightly decreased compared to the baseline value (T1), and the concentration of 2‐AG stagnates (Table 2). Further, only the concentration of AEA at T2 approached the concentration of AEA in the group of healthy participants (Table 3).

**TABLE 3:** Comparison of the endocannabinoid concentration and psychometry of patients at the second appointment (T2) and healthy participants.

	Patients (T2)	Healthy participants
Number of samples	57	
Endocannabinoids		
AEA (nM) (*n* = 57) (M ± SD)	0.986 ± 0.704	0.823 ± 0.427
*t*‐Test	*t*(112) = 1.49, *p* = .139	
2‐AG (nM) (*n* = 54) (M ± SD)	2.75 ± 1.66	4.10 ± 2.27
*t*‐Test	*t*(97.19) = −3.52, *p* = .001***	
Psychometry		
HAMD‐21 (*n* = 57) (M ± SD)	16.94 ± 17.31	0.684 ± 1.09
*t*‐Test	*t*(56.44) = 7.08, *p* = .000 ***	
BDI‐II (*n* = 57) (M ± SD)	28.75 ± 18.65	1.91 ± 2.76
*t*‐Test	*t*(58.45) = 10.75, *p* = .000***	
STAI‐X1 (*n* = 57) (M ± SD)	55.32 ± 16.45	30.32 ± 6.10
*t*‐Test	*t*(71.13) = 10.76, *p* = .000***	
STAI‐X2 (*n* = 57) (M ± SD)	56.86 ± 14.62	30.59 ± 6.04
*t*‐Test	*t*(57.26) = 11.02, *p* = .000***	

Abbreviations: 2‐AG, 2‐arachidonoylglycerol; AEA, arachidonoyl ethanolamide; BDI‐II, Beck depression inventory; HAMD‐21, Hamilton; M, mean value; SD, standard deviation; STAI‐X1, state anxiety inventory; STAI‐X2, trait anxiety inventory.

***p < .001.

Additional analysis showed a negative correlation between the content of AEA and the self‐questionnaire STAI‐X1 (*r* = −.267, *p* = .023) only in the group of patients with a depressive disorder. There was no correlation between endocannabinoids (AEA, 2‐AG) and the other self‐questionnaires. Furthermore, a gender difference was found concerning the 2‐AG concentration in patients. Accordingly, male patients showed higher concentrations of 2‐AG compared to female patients (male (*n* = 28) = 3.33 ± 1.45 nM; female (*n* = 41) = 2.63 ± 1.35 nM; *t*(67) = 2.07; *p* = .043). In the group of healthy controls, there was no gender difference.

### 4.3 Genotyping and differences between the genotypes

As several polymorphisms are known to increase the likelihood of developing depression, we analyzed the genotypes of the 5‐HT1A (*rs6295)*, 5‐HT2A (*rs6311*), and the CNR1 (*rs1049353*) genes to explore a possible link between alterations in these genes and depression.

The genotypic distributions of the three polymorphisms were all in Hardy–Weinberg equilibrium in the patients and group of healthy participants (see Table 4). The results of 5‐HT1A, 5‐HT2A, and CNR1 genotyping are shown in Table 4. There was no significant difference between the distributions of the various genotypes between the patients and healthy participants.

**TABLE 4:** Genotyping of the 5‐HT1A, 5‐HT2A, and CNR1 polymorphism in patients with depression and healthy participants.

Gene	Group	Number of alleles (frequency)			*χ* ^2^	*p*‐Value
5‐HT1A *rs6295*		GG	GC	CC	.562	.755
	Patients (*n* = 154) (*n*/%)	45 (29.2)	75 (48.7)	34 (22.1)		
	Healthy participants (*n* = 154) (*n*/%)	51 (33.1)	70 (45.5)	33 (21.4)		
5‐HT2A *rs6311*		GG	GA	AA	4.112	.128
	Patients (*n* = 158) (*n*/%)	54 (34.2)	70 (44.3)	34 (21.5)		
	Healthy participants (*n* = 158) (*n*/%)	38 (24.1)	84 (53.2)	36 (22.8)		
CNR1 *rs1049353*		GG	GA	AA	2.236	.327
	Patients (*n* = 153) (*n*/%)	76 (49.7)	62 (40.5)	15 (9.8)		
	Healthy participants (*n* = 153) (*n*/%)	76 (49.7)	54 (35.3)	23 (15.0)		

In a previous study, compared to healthy participants, we examined the relationship between serotonergic activity (cortical: patients 0.256 ± 0.186/healthy participants 0.252 ± 0.119; *p* = .855) and the concentration of serotonin in the peripheral blood (serum (patient: 230.9 ± 326.0 ng/mL/healthy participants: 773.8 ± 646.0 ng/mL; *p* < .001)/platelet (patient: 1113.9 ± 964.1 10^9^/Thr/healthy participants: 2008.8 ± 1026.4 10^9^/Thr; *p* < .001)) in patients with depression (see Obermanns et al., 2022). Concerning the CNR1 gene, there was a difference among the homozygote GG carriers, the AA allele genotype carriers, and the BDI‐II in the group of healthy participants. Healthy participants who are homozygote for GG reached higher scores in the BDI‐II than homozygote AA carriers (GG (*n* = 79) = 3.48 ± 4.10; AA (*n* = 23) = 1.26 ± 1.81; *t*(83.862) = 3.719, *p* = .000). Furthermore, healthy participants who are CNR1 GA carriers showed increased concentration of the endocannabinoid 2‐AG compared to GG carriers (GA (*n* = 19) = 5.05 nM ± 2.49 nM; GG (*n* = 32) = 3.51 nM ± 1.92 nM; *t*(49) = −2.475, *p* = .017). These results could not be detected in patients. In contrast, some distinctions concerning the 5‐HT1A and 5‐HT2A genotypes could be determined only in the group of patients. Relating to the 5‐HT1A gene, patients with a homozygote variant of the C allele reached higher values in the cortical LDAEP than patients with the heterozygote variant GC (CC (*n* = 16) = 0.339 ± 0.215; GC (*n* = 32) = 0.198 ± 0.140; *t*(21.610) = 2.384, *p* = .026). Not only in the cortical LDAEP distinctions but also in the source LDAEP (left hemisphere). The reached values differ between the genotypes of the 5‐HT1A. In a comparison of homozygote C and G carriers with the heterozygote GC carriers, the homozygous carriers showed higher values of the source LDAEP in the left hemisphere (CC (*n* = 16) = 0.292 ± 0.202; GC (*n* = 32) = 0.126 ± 0.116; *t*(20.058) = 3.043, *p* = .006); GG (*n* = 18) = 0.276 ± 0.270; *t*(20.577) = 2.247, *p* = .036). Additionally, the serotonin serum content in patients who carry the GG variant of the 5‐HT2A gene is increased compared to AA carriers (GG (*n* = 28) = 385.55 ng/mL ± 458.01 ng/mL; AA (*n* = 19) = 139.78 ng/mL ± 176.09 ng/mL; *t*(37.383) = 2.573, *p* = .014).

## 5 DISCUSSION

### 5.1 Altered concentration of endocannabinoid AEA and 2‐AG in depression

In this study, we could examine a decreased concentration of 2‐AG and a higher concentration of AEA in patients with a depressive disorder compared to healthy participants. These findings are partially consistent with studies that reported altered endocannabinoid levels in depressive disorders (Hill, Miller, et al., 2008; Hill et al., 2009; Behnke et al., 2022; Garani et al., 2021; Romero‐Sanchiz et al., 2019). In contrast to the above studies, Coccaro et al. (2018) did not find a significant change in circulating endocannabinoids. It cannot be fundamentally assumed that hypofunction is present in depressive disorder. Differences in endocannabinoid concentrations may be due to differences in cohort gender composition. In our study, we were able to detect a gender difference between male and female patients. Male patients showed an increased concentration of 2‐AG compared to female patients. Meanwhile, it is now known that gender‐specific differences exist in the endocannabinoid system (Tabatadze et al., 2015); for example, the influence of sex hormones on the function of the endocannabinoid system (Struik et al., 2018) or the different availabilities of CBR1 receptors in the brain (Laurikainen et al., 2019). According to Cedernaes et al. (2016), endocannabinoids can pass the blood–brain barrier, which could suggest that the peripheral endocannabinoid content (AEA, 2‐AG) may represent the central endocannabinoid content. However, there is no direct correlation or explicit relationship between peripheral and central endocannabinoid levels (Giuffrida et al., 2004). Further investigation would be needed to explore this hypothesis.

Interestingly, this study found a negative correlation between anxiety symptoms and AEA levels in depressed patients, consistent with findings from other studies (Bluett et al., 2014; Green et al., 2022). Coccaro et al. (2018), Hill, Miller, et al. (2008), Dlugos et al. (2012) did not find a significant correlation between AEA and anxiety as a condition. A reduction in AEA can be triggered by stress in limbic brain regions and inhibition of AEA signaling, which can lead to anxiety symptoms (Hill et al., 2013; Hill and Gorzalka, 2005). In contrast to reduced AEA signaling during a stress response, 2‐AG signaling is increased. This increase is mediated by corticosterone. Both lead to the termination of the stress response and limit the effects of stress on the brain (Morena et al., 2016; Yin et al., 2019). Mental illnesses such as depression, usually accompanied by anxiety, cause stress. The ECS responds by adapting to the changes and regulating the concentrations of AEA and 2‐AG. Further, a reduced concentration of AEA can lead to anxiety‐like behavior. By inhibiting the enzyme fatty acid amide hydrolase (FAAH), the concentration of AEA increases, and anxious symptoms decrease (Bluett et al., 2014; Green et al., 2022). Conversely, this means that the inhibition of the enzyme FAAH could induce anxiolytic effects and may help treat depression, which is associated with increased anxiety.

From these findings, the endocannabinoid system may be involved in the psychopathology of depression, but other factors, such as stress, medication, or sex (Garani et al., 2021; Romero‐Sanchiz et al., 2019; Tabatadze et al., 2015) can influence the endocannabinoid system, especially the amount of AEA and 2‐AG and should be considered.

Furthermore, we also investigated the variation of the endocannabinoid level of AEA and 2‐AG, as well as symptoms of depression and anxiety during the treatment in patients with a depressive disorder. We observed a reduction in symptoms of depression and a stagnation in anxiety symptoms. The level of AEA and 2‐AG changed not significantly during the treatment.

No comparable study exists in the literature that has measured changes in circulating endocannabinoid concentrations in peripheral blood. In human studies, changes in the concentration of AEA and 2‐AG could be observed under the treatment of electroconvulsive therapy and repetitive transcranial magnetic stimulation (Kranaster et al., 2017; Lazary et al., 2021). An animal study also found a change in endocannabinoid concentrations after 21 days (Hill, Carrier, et al., 2008). The comparability and transferability to the results of this work are questionable, as in the study of Kranaster et al., 2017 the concentrations of endocannabinoids were measured from the cerebrospinal fluid and not in the peripheral blood. So far, there are no correlations between peripheral and central endocannabinoid concentrations (Giuffrida et al., 2004), which means that no conclusions can be drawn about peripheral concentrations.

The present finding could reveal an improvement in the severity of depression during the treatment. This indicates that the severity of depression can be reduced within 28 days, but not the severity of anxiety and an alteration in the level of endocannabinoids. Here, future studies are necessary to investigate the time course of responsiveness of the endocannabinoid system during the treatment.

### 5.2 Genetic variation of the 5‐HT1A, 5‐HT2A, and CNR1 gene

The influence of genetic factors on the etiology of depression cannot be excluded. The possible effects of the polymorphism in the genes of the CNR1 (rs1040353), 5‐HT1A (rs6295), and 5‐HT2A (rs6311), which are already related to depression, were investigated in this study.

The genetic distribution of the CNR1, 5‐HT1A, and 5‐HT2A showed no significant differences between patients with depression and healthy participants.

Regarding the polymorphism in the CNR1 gene, not many studies have investigated the distribution of the CNR1 rs1049353 in patients with depression. Referring to this, controversial results are published. Some studies could show a link between the CNR1 rs1049353 polymorphism and depression, and others not (Domschke et al., 2008; Juhasz et al., 2009; Kong et al., 2019; Monteleone et al., 2010). The haplotypic combination of the A‐allele seems to be associated with an increased risk for neuroticism and depression (Juhasz et al., 2009). In contrast, Schennach et al. (2012) reported a link between the G allele with higher depressive‐like symptoms. In this study, an association of the CNR1 rs104353 polymorphism with depression was not found in patients with depression.

Interestingly, we found differences between depressive symptoms (BDI‐II) and homozygote CC and GG carriers in healthy participants. We cannot explain why these differences only exist in healthy participants and not in patients. Further, healthy participants who are homozygote for the G allele showed decreased 2‐AG levels compared to heterozygote GG carriers. To our knowledge, no recent study has investigated the impact of the CNR1 rs1049353 polymorphism on the 2‐AG level in healthy participants. Further research is advisable to explain the relationship among the different genotypes of the CNR1 rs1049353 polymorphism and the level of endocannabinoids in healthy participants.

Besides the endocannabinoid receptor polymorphism CNR1 rs1049353, the polymorphism 5‐HT1A rs6295 and 5‐HT2A rs6311 could play a crucial role in the etiology of depression. As mentioned above, we found no significant difference in the allelic variation between patients and healthy participants.

Nevertheless, in this study, a difference among the different genotypes of the 5‐HT1A rs6295 polymorphism was found concerning the serotonergic activity in the brain of patients. Homozygous patients for both the G and C alleles showed lower serotonergic activity than heterozygous carriers. Previous studies have suggested that 5‐HT1A receptors may be involved in the serotonergic modulation of the loudness‐dependent response in the primary auditory cortex (Guille et al., 2011). Although the mechanisms leading to the inverse relationship of LDAEP (serotonergic activity) and serotonin levels are not fully elucidated, the activation of 5‐HT1A receptors is thought to be due to pyramidal cells (Puig et al., 2005). Furthermore, a link between the LDAEP and 5‐HT1A receptors in depression has been reported (Pillai et al., 2020). Albert (2012) was able to link altered 5‐HT1A receptor function and expression to the rs6295 polymorphism. When the G allele was present, it prevented the repressor from binding to DNA. This resulted in an increase in 5‐HT1A autoreceptors and decreased serotonergic neurotransmission. Further, several studies suggest an association between the allelic variation of the 5‐HT1A receptor and the risk of developing depression (Biard et al., 2016; Albert et al., 2019; Kaufman et al., 2016). It was hypothesized that the rs6295 polymorphism is enabled to regulate the expression of the 5‐HT1A genes in neurons of the raphe (Albert & Lemonade, 2004). Individuals carrying the G allele cannot bind to the transcription factors NUDR/DEAF‐1; the resulting transcriptional repression leads to an overexpression of the 5‐HT1A autoreceptor in the raphe compared to homozygous C allele carriers. This leads to reduced serotonergic firing and consequently to reduce serotonergic activity, which is associated with depression (see review Kaufman et al., 2016 and Albert et al., 2019). Our results are partially consistent with previous findings. The reduced serotonergic activity compared to heterozygotes was observed in the patient's homozygotes for the G allele. However, a lower activity in homozygote C carriers compared to heterozygotes was observed. We do not know of any study in which the same results were obtained regarding reduced serotonergic activity in homozygotic CC and GG allele carriers compared to heterozygous carriers. It seems that the homozygote variants (GG/CC) of the 5‐HT1A polymorphism may lead to altered serotonergic activity in the brains of depressed patients.

When focusing on the association between the 5‐HT2A rs6311 polymorphism and depression, we found increased 5‐HT serum levels in patients who are homozygote for the G allele compared to homozygote A allele carriers. To our knowledge, the relation between serum serotonin concentration and genetic variations of the 5‐HT2A receptor in patients with depression has not been investigated. It is difficult to explain this finding because most studies have focused on the 5‐HT2A rs6311 polymorphism concerning platelet aggregation. Despite a lack of literature regarding serum serotonin concentration and 5‐HT2A, studies exist suggesting that this polymorphism is a susceptibility factor in the etiology of depression (Cao et al., 2015; Pandey et al., 2010). Other studies report the opposite (Hong et al., 2006; Jin et al., 2013; Kang et al., 2007). It is well known that mutations in this gene may alter promoter activity and 5‐HT2A receptor expression (Parsons et al., 2004). In platelets, genetic variation in the regulatory region of the 5‐HT2A receptor gene could lead to altered levels of 5‐HT2A receptors. Despite these findings, no conclusions regarding the decreased serotonin concentration in homozygous A allele carriers can be drawn. Further studies with a larger sample size are advised to verify these results and to investigate the influence of genetic variation of the 5‐HT2A receptor on serotonin serum concentration.

The recent results of this study have demonstrated and supported a possible association between the genetic variations of the 5‐HT1A rs6395 and 5‐HT2A rs6311 polymorphisms and an altered serotonergic system in patients with depressive disorder. Results on genetic variations and their associations should be taken into account and cautiously consider the small number of samples and the influence of possible environmental risk factors, such as stressful life events. A larger number of samples are needed to make more accurate statements about the influence of genetic factors.

## 6 LIMITATION

Our study has several limitations. For one, as we included an uneven number of medicated and unmedicated patients, our assumptions cannot be generalized because the treatment with antidepressants may affect the level of endocannabinoids (Hill, Hillard, et al., 2009; Romero‐Sanchiz et al., 2019). We should have included more unmedicated patients in the study to draw more accurate conclusions about altered endocannabinoid concentration in patients with depressive disorder. Second, the sample size of the study cohort was relatively small for genetic associations. To strengthen the power of genotyping, the results of this study should be replicated in a larger study cohort. Third, we should have established additional questionnaires, which assess stressful events, as previous investigations suggest a possible influence on the serotonergic‐ and endocannabinoid system, as well as on the development of depression (Morena et al., 2016; Navarro et al., 2022).

Fourth, the method for endocannabinoid determination should be established before the first samples are collected. This would ensure a rapid processing of samples, avoiding an elimination of the analytes before the analysis and verifying data a priori. With the current analytical methodology, a batch‐to‐batch consistency with isotope‐labeled internal standards is easier to maintain than when different samples are measured in one batch after partially extended storage periods. Even after establishing and optimizing the method, no endocannabinoids could be measured in one subcohort, indicating the need for an advanced analytical study design. Possible causes could be storage time and temperature fluctuations during sample transport and handling, as active catabolism can occur for this compound class, and endocannabinoids adsorb to plastics and glassware, which can lead to analyte losses but also in‐built limitations of the analytical system in use (Karlsson et al., 2004; Kratz et al., 2021; Rouzer et al., 2002; Schreiber et al., 2007; Wood et al., 2008).

## 7 CONCLUSION

In conclusion, the present results showed altered concentrations of the endocannabinoids AEA and 2‐AG in patients with depression compared to healthy participants. Furthermore, after treatment (T1/T2), an improvement of depressive symptoms in patients with a depressive disorder has been observed. Based on genotyping, no different distributions of the CNR1, 5‐Ht1A, and 5‐HT2A genes were found in patients with depressive disorder and healthy participants. However, certain genotypes of the 5‐HT1A (rs6295) and 5‐HT2A (rs6311) polymorphisms were shown to have different effects on the serotonergic system in patients. In controls, the different genotypes of the CNR1 rs4910353 polymorphism were associated with 2‐AG levels and depressive symptoms. Furthermore, in patients, there is a correlation between AEA concentration and anxiety symptoms.

More investigations concerning the impact of genetic variations on the serotonergic and endocannabinoid systems in terms of involvement in the development of depression are advisable. In summary, our results suggest an altered endocannabinoid system in patients with a depressive disorder and a possible influence of the polymorphism in the CNR1, 5‐HT1A, and 5‐HT2A genes.

## AUTHOR CONTRIBUTIONS

**Jasmin Obermanns**: Conceptualization; data curation; investigations; formal analysis; methodology; validation; writing—original draft. **Hanna Meiser**: Data curation; investigations; writing—review and editing. **Saskia Hoberg**: Data curation; investigations; writing—review and editing. **Cynthia Segura Vesterager**: Investigations; methodology; writing—review and editing. **Frank Schulz**: Writing—review and editing. **Georg Juckel**: Project administration; supervision; writing—review and editing. **Barbara Emons**: Conceptualization; funding acquisition; project administration; supervision; writing—review and editing. All authors approved the final version of the paper.

## CONFLICT OF INTEREST STATEMENT

The authors declare no conflicts of interest.

### PEER REVIEW

The peer review history for this article is available at https://publons.com/publon/10.1002/brb3.3323.

## Data Availability

The original data files are available by contacting the first or last author.

## References

[brb33323-bib-0001] Ackenheil, M. , Stotz, G. , Dietz‐Bauer, R. , & Vossen, A. (1999). Deutsche Fassung des Mini—International Neuropsychiatric Interview. Psychiatrische Universitätsklinik München.

[brb33323-bib-0002] Albert, P. R. (2012). Transcriptional regulation of the 5‐HT1A receptor: Implications for mental illness. Philosophical Transactions of the Royal Society B: Biological Sciences, 367(1601), 2402–2415. 10.1098/rstb.2011.0376 PMC3405675 22826341

[brb33323-bib-0003] Albert, P. R. , Le François, B. , & Vahid‐Ansari, F. (2019). Genetic, epigenetic and posttranscriptional mechanisms for treatment of major depression: The 5‐HT1A receptor gene as a paradigm. Journal of Psychiatry and Neuroscience, 44(3), 164–176. 10.1503/jpn.180209 30807072 PMC6488484

[brb33323-bib-0004] Albert, P. R. , & Lemonde, S. (2004). 5‐HT1A receptors, gene repression, and depression: Guilt by association. The Neuroscientist, 10(6), 575–593. 10.1177/1073858404267382 15534042

[brb33323-bib-0006] Behnke, A. , Gumpp, A. M. , Rojas, R. , Sänger, T. , Lutz‐Bonengel, S. , Moser, D. , Schelling, G. , Krumbholz, A. , & Kolassa, I.‐T. (2022). Circulating inflammatory markers, cell‐free mitochondrial DNA, cortisol, endocannabinoids, and *N*‐acylethanolamines in female depressed outpatients. The World Journal of Biological Psychiatry: The Official Journal of the World Federation of Societies of Biological Psychiatry, 24, 58–69. Advance online publication. 10.1080/15622975.2022.2070666 35532037

[brb33323-bib-0007] Biard, K. , Douglass, A. B. , Robillard, R. , & De Koninck, J. (2016). A pilot study of serotonin‐1A receptor genotypes and rapid eye movement sleep sensitivity to serotonergic/cholinergic imbalance in humans: A pharmacological model of depression. Nature and Science of Sleep, 8, 1. 10.2147/NSS.S94549 PMC4690650 26719734

[brb33323-bib-0008] Bluett, R. J. , Gamble‐George, J. C. , Hermanson, D. J. , Hartley, N. D. , Marnett, L. J. , & Patel, S. (2014). Central anandamide deficiency predicts stress‐induced anxiety: Behavioral reversal through endocannabinoid augmentation. Translational Psychiatry, 4(7), e408–e408. 10.1038/tp.2014.53 25004388 PMC4119220

[brb33323-bib-0009] Bright, U. , & Akirav, I. (2022). Modulation of endocannabinoid system components in depression: Pre‐clinical and clinical evidence. International Journal of Molecular Sciences, 23(10), 5526. 10.3390/ijms23105526 35628337 PMC9146799

[brb33323-bib-0010] Busch, Y. , & Menke, A. (2019). Blood‐based biomarkers predicting response to antidepressants. Journal of Neural Transmission (Vienna, Austria: 1996), 126(1), 47–63. 10.1007/s00702-018-1844-x 29374800

[brb33323-bib-0011] Cao, S. , Li, H. , Lou, L. , Xie, Z. , Zhao, X. , Pang, J. , Sui, J. , & Xie, G. (2015). Association study between 5‐HT 2A and NET gene polymorphisms and recurrent major depression disorder in Chinese Han population. Pakistan Journal of Pharmaceutical Sciences, 28, 1101–1108. 26051731

[brb33323-bib-0012] Cedernaes, J. , Fanelli, F. , Fazzini, A. , Pagotto, U. , Broman, J.‐E. , Vogel, H. , Dickson, S. L. , Schiöth, H. B. , & Benedict, C. (2016). Sleep restriction alters plasma endocannabinoids concentrations before but not after exercise in humans. Psychoneuroendocrinology, 74, 258–268. 10.1016/j.psyneuen.2016.09.014 27689899

[brb33323-bib-0013] Celada, P. , Puig, M. V. , Amargós‐Bosch, M. , Adell, A. , & Artigas, F. (2004). The therapeutic role of 5‐HT1A and 5‐HT2A receptors in depression. Journal of Psychiatry and Neuroscience, 29(4), 252–265. 15309042 PMC446220

[brb33323-bib-0014] Choi, K. , Le, T. , Mcguire, J. , Xing, G. , Zhang, L. , Li, H. , Parker, C. C. , Johnson, L. R. , & Ursano, R. J. (2012). Expression pattern of the cannabinoid receptor genes in the frontal cortex of mood disorder patients and mice selectively bred for high and low fear. Journal of Psychiatric Research, 46(7), 882–889. 10.1016/j.jpsychires.2012.03.021 22534181

[brb33323-bib-0016] Coccaro, E. F. , Hill, M. N. , Robinson, L. , & Lee, R. J. (2018). Circulating endocannabinoids and affect regulation in human subjects. Psychoneuroendocrinology, 92, 66–71. 10.1016/j.psyneuen.2018.03.009 29627714

[brb33323-bib-0017] Dlugos, A. , Childs, E. , Stuhr, K. L. , Hillard, C. J. , & De Wit, H. (2012). Acute stress increases circulating anandamide and other *N‐acylethanolamines* in healthy humans. Neuropsychopharmacology, 37, 2416–2427. 10.1038/npp.2012.100 22763622 PMC3442338

[brb33323-bib-0018] Domschke, K. , Braun, M. , Ohrmann, P. , Suslow, T. , Kugel, H. , Bauer, J. , Hohoff, C. , Kersting, A. , Engelien, A. , Arolt, V. , Heindel, W. , & Deckert, J. (2006). Association of the functional ‐1019C/G 5‐HT1A polymorphism with prefrontal cortex and amygdala activation measured with 3 T fMRI in panic disorder. The International Journal of Neuropsychopharmacology, 9(3), 349–355. 10.1017/S1461145705005869 16316476

[brb33323-bib-0019] Domschke, K. , Dannlowski, U. , Ohrmann, P. , Lawford, B. , Bauer, J. , Kugel, H. , Heindel, W. , Young, R. , Morris, P. , Arolt, V. , Deckert, J. , Suslow, T. , & Baune, B. T. (2008). Cannabinoid receptor 1 (CNR1) gene: Impact on antidepressant treatment response and emotion processing in major depression. European Neuropsychopharmacology, 18(10), 751–759. 10.1016/j.euroneuro.2008.05.003 18579347

[brb33323-bib-0020] Fakra, E. , Hyde, L. W. , Gorka, A. , Fisher, P. M. , Muñoz, K. E. , Kimak, M. , Halder, I. , Ferrell, R. E. , Manuck, S. B. , & Hariri, A. R. (2009). Effects of HTR1A C(‐1019)G on amygdala reactivity and trait anxiety. Archives of General Psychiatry, 66(1), 33–40. 10.1001/archpsyc.66.1.33 19124686 PMC2736132

[brb33323-bib-0021] Garani, R. , Watts, J. J. , & Mizrahi, R. (2021). Endocannabinoid system in psychotic and mood disorders, a review of human studies. Progress in Neuro‐Psychopharmacology and Biological Psychiatry, 106, 110096. 10.1016/j.pnpbp.2020.110096 32898588 PMC8582009

[brb33323-bib-0022] Giuffrida, A. , Leweke, F. M. , Gerth, C. W. , Schreiber, D. , Koethe, D. , Faulhaber, J. , Klosterkötter, J. , & Piomelli, D. (2004). Cerebrospinal anandamide levels are elevated in acute schizophrenia and are inversely correlated with psychotic symptoms. Neuropsychopharmacology, 29(11), 2108–2114. 10.1038/sj.npp.1300558 15354183

[brb33323-bib-0023] Green, D. G. , Westwood, D. J. , Kim, J. , Best, L. M. , Kish, S. J. , Tyndale, R. F. , Mccluskey, T. , Lobaugh, N. J. , & Boileau, I. (2022). Fatty acid amide hydrolase levels in brain linked with threat‐related amygdala activation. Neuroimage: Reports, 2(2), 100094. 10.1016/j.ynirp.2022.100094 37235067 PMC10206405

[brb33323-bib-0024] Guille, V. , Gogos, A. , Nathan, P. J. , Croft, R. J. , & Van Den Buuse, M. (2011). Interaction of estrogen with central serotonergic mechanisms in human sensory processing: Loudness dependence of the auditory evoked potential and mismatch negativity. Journal of Psychopharmacology (Oxford, England), 25(12), 1614–1622. 10.1177/0269881110370506 20562170

[brb33323-bib-0026] Hamilton, M. (1960). A rating scale for depression. Journal of Neurology, Neurosurgery, and Psychiatry, 23(1), e56. 10.1136/jnnp.23.1.56 PMC495331 14399272

[brb33323-bib-0027] Häring, M. , Enk, V. , Aparisi Rey, A. , Loch, S. , Ruiz De Azua, I. , Weber, T. , Bartsch, D. , Monory, K. , & Lutz, B. (2015). Cannabinoid type‐1 receptor signaling in central serotonergic neurons regulates anxiety‐like behavior and sociability. Frontiers in Behavioral Neuroscience, 9, 235. 10.3389/fnbeh.2015.00235 26388750 PMC4558975

[brb33323-bib-0028] Hautzinger, M. , Keller, F. , & Kühner, C. (2006). Beck depressions‐inventar (BDI‐II). Harcourt TestServices GmbH.

[brb33323-bib-0030] Hill, M. , Miller, G. , Ho, W.‐S. , Gorzalka, B. , & Hillard, C. (2008). Serum endocannabinoid content is altered in females with depressive disorders: A preliminary report. Pharmacopsychiatry, 41(2), 48–53. 10.1055/s-2007-993211 18311684 PMC3422568

[brb33323-bib-0031] Hill, M. N. , Carrier, E. J. , Mclaughlin, R. J. , Morrish, A. C. , Meier, S. E. , Hillard, C. J. , & Gorzalka, B. B. (2008). Regional alterations in the endocannabinoid system in an animal model of depression: Effects of concurrent antidepressant treatment. Journal of Neurochemistry, 106(6), 2322–2336. 10.1111/j.1471-4159.2008.05567.x 18643796 PMC2606621

[brb33323-bib-0032] Hill, M. N. , & Gorzalka, B. B. (2005). Is there a role for the endocannabinoid system in the etiology and treatment of melancholic depression? Behavioural Pharmacology, 16(5–6), 333–352. 10.1097/00008877-200509000-00006 16148438

[brb33323-bib-0033] Hill, M. N. , Hillard, C. J. , Bambico, F. R. , Patel, S. , Gorzalka, B. B. , & Gobbi, G. (2009). The therapeutic potential of the endocannabinoid system for the development of a novel class of antidepressants. Trends in Pharmacological Sciences, 30(9), 484–493. 10.1016/j.tips.2009.06.006 19732971

[brb33323-bib-0034] Hill, M. N. , Kumar, S. A. , Filipski, S. B. , Iverson, M. , Stuhr, K. L. , Keith, J. M. , Cravatt, B. F. , Hillard, C. J. , Chattarji, S. , & Mcewen, B. S. (2013). Disruption of fatty acid amide hydrolase activity prevents the effects of chronic stress on anxiety and amygdalar microstructure. Molecular Psychiatry, 18(10), 1125–1135. 10.1038/mp.2012.90 22776900 PMC4148304

[brb33323-bib-0035] Hill, M. N. , Mclaughlin, R. J. , Morrish, A. C. , Viau, V. , Floresco, S. B. , Hillard, C. J. , & Gorzalka, B. B. (2009). Suppression of amygdalar endocannabinoid signaling by stress contributes to activation of the hypothalamic–pituitary–adrenal axis. Neuropsychopharmacology, 34(13), 2733–2745. 10.1038/npp.2009.114 19710634 PMC3197779

[brb33323-bib-0036] Hill, M. N. , Miller, G. E. , Carrier, E. J. , Gorzalka, B. B. , & Hillard, C. J. (2009). Circulating endocannabinoids and *N*‐acyl ethanolamines are differentially regulated in major depression and following exposure to social stress. Psychoneuroendocrinology, 34(8), 1257–1262. 10.1016/j.psyneuen.2009.03.013 19394765 PMC2716432

[brb33323-bib-0037] Hong, C.‐J. , Chen, T.‐J. , Yu, Y. W.‐Y. , & Tsai, S.‐J. (2006). Response to fluoxetine and serotonin 1A receptor (C‐1019G) polymorphism in Taiwan Chinese major depressive disorder. The Pharmacogenomics Journal, 6(1), 27–33. 10.1038/sj.tpj.6500340 16302021

[brb33323-bib-0038] Ibarra‐Lecue, I. , Mollinedo‐Gajate, I. , Meana, J. J. , Callado, L. F. , Diez‐Alarcia, R. , & Urigüen, L. (2018). Chronic cannabis promotes pro‐hallucinogenic signaling of 5‐HT2A receptors through Akt/mTOR pathway. Neuropsychopharmacology, 43(10), 2028–2035. 10.1038/s41386-018-0076-y 29748632 PMC6098160

[brb33323-bib-0039] Ibarra‐Lecue, I. , Pilar‐Cuéllar, F. , Muguruza, C. , Florensa‐Zanuy, E. , Díaz, Á. , Urigüen, L. , Castro, E. , Pazos, A. , & Callado, L. F. (2018). The endocannabinoid system in mental disorders: Evidence from human brain studies. Biochemical Pharmacology, 157, 97–107. 10.1016/j.bcp.2018.07.009 30026022

[brb33323-bib-0040] Jin, C. , Xu, W. , Yuan, J. , Wang, G. , & Cheng, Z. (2013). Meta‐analysis of association between the‐1438A/G (rs6311) polymorphism of the serotonin 2A receptor gene and major depressive disorder. Neurological Research, 35(1), 7–14. 10.1179/1743132812Y.0000000111 23317793

[brb33323-bib-0041] Juhasz, G. , Chase, D. , Pegg, E. , Downey, D. , Toth, Z. G. , Stones, K. , Platt, H. , Mekli, K. , Payton, A. , Elliott, R. , Anderson, I. M. , & Deakin, J. F. W. (2009). CNR1 gene is associated with high neuroticism and low agreeableness and interacts with recent negative life events to predict current depressive symptoms. Neuropsychopharmacology, 34(8), 2019–2027. 10.1038/npp.2009.19 19242408

[brb33323-bib-0043] Kang, R.‐H. , Choi, M.‐J. , Paik, J.‐W. , Hahn, S.‐W. , & Lee, M.‐S. (2007). Effect of serotonin receptor 2A gene polymorphism on mirtazapine response in major depression. International Journal of Psychiatry in Medicine, 37(3), 315–329. 10.2190/PM.37.3.h 18314859

[brb33323-bib-0044] Karlsson, M. , Påhlsson, C. , & Fowler, C. J. (2004). Reversible, temperature‐dependent, and AM404‐inhibitable adsorption of anandamide to cell culture wells as a confounding factor in release experiments. European Journal of Pharmaceutical Sciences: Official Journal of the European Federation for Pharmaceutical Sciences, 22(2–3), 181–189. 10.1016/j.ejps.2004.03.009 15158903

[brb33323-bib-0045] Kato, M. , Fukuda, T. , Wakeno, M. , Fukuda, K. , Okugawa, G. , Ikenaga, Y. , Yamashita, M. , Takekita, Y. , Nobuhara, K. , Azuma, J. , & Kinoshita, T. (2006). Effects of the serotonin type 2A, 3A and 3B receptor and the serotonin transporter genes on paroxetine and fluvoxamine efficacy and adverse drug reactions in depressed Japanese patients. Neuropsychobiology, 53(4), 186–195. 10.1159/000094727 16874005

[brb33323-bib-0046] Kaufman, J. , Delorenzo, C. , Choudhury, S. , & Parsey, R. V. (2016). The 5‐HT1A receptor in major depressive disorder. European Neuropsychopharmacology, 26(3), 397–410. 10.1016/j.euroneuro.2015.12.039 26851834 PMC5192019

[brb33323-bib-0047] Kim, H. K. , Kim, S. J. , Lee, Y. J. , Lee, H.‐J. , Kang, S.‐G. , Choi, J.‐E. , Yun, K.‐W. , & Lim, W.‐J. (2011). Influence of the interaction between the serotonin 1A receptor C‐1019G polymorphism and negative life stressors on the development of depression. Neuropsychobiology, 64(1), 1–8. 10.1159/000322144 21577007

[brb33323-bib-0048] Kishi, T. , Tsunoka, T. , Ikeda, M. , Kawashima, K. , Okochi, T. , Kitajima, T. , Kinoshita, Y. , Okumura, T. , Yamanouchi, Y. , Inada, T. , Ozaki, N. , & Iwata, N. (2009). Serotonin 1A receptor gene and major depressive disorder: An association study and meta‐analysis. Journal of Human Genetics, 54(11), 629–633. 10.1038/jhg.2009.84 19730445

[brb33323-bib-0049] Köhler, S. , Cierpinsky, K. , Kronenberg, G. , & Adli, M. (2016). The serotonergic system in the neurobiology of depression: Relevance for novel antidepressants. Journal of Psychopharmacology, 30(1), 13–22. 10.1177/0269881115609072 26464458

[brb33323-bib-0050] Kong, X. , Miao, Q. , Lu, X. , Zhang, Z. , Chen, M. , Zhang, J. , & Zhai, J. (2019). The association of endocannabinoid receptor genes (CNR1 and CNR2) polymorphisms with depression: A meta‐analysis. Medicine, 98(46), e17403. 10.1097/MD.0000000000017403 31725603 PMC6867758

[brb33323-bib-0051] Kranaster, L. , Hoyer, C. , Aksay, S. S. , Bumb, J. M. , Leweke, F. M. , Janke, C. , Thiel, M. , Lutz, B. , Bindila, L. , & Sartorius, A. (2017). Electroconvulsive therapy enhances endocannabinoids in the cerebrospinal fluid of patients with major depression: A preliminary prospective study. European Archives of Psychiatry and Clinical Neuroscience, 267(8), 781–786. 10.1007/s00406-017-0789-7 28342110

[brb33323-bib-0052] Kratz, D. , Thomas, D. , & Gurke, R. (2021). Endocannabinoids as potential biomarkers: It's all about pre‐analytics. Journal of Mass Spectrometry and Advances in the Clinical Lab, 22, 56–63. 10.1016/j.jmsacl.2021.11.001 34939056 PMC8662329

[brb33323-bib-0053] Laurikainen, H. , Tuominen, L. , Tikka, M. , Merisaari, H. , Armio, R.‐L. , Sormunen, E. , Borgan, F. , Veronese, M. , Howes, O. , Haaparanta‐Solin, M. , Solin, O. , & Hietala, J. (2019). Sex difference in brain CB1 receptor availability in man. Neuroimage, 184, 834–842. 10.1016/j.neuroimage.2018.10.013 30296558

[brb33323-bib-0054] Laux, L. , Glanzmann, P. , Schaffner, P. , & Spielberger, C. D. (1981). Das State‐Trait‐Angstinventar. Theoretische Grundlagen und Handanweisung. Beltz Test GmbH.

[brb33323-bib-0055] Lazary, J. , Elemery, M. , Dome, P. , Kiss, S. , Gonda, X. , Tombor, L. , Pogany, L. , Becskereki, G. , Toth, B. , & Faludi, G. (2021). Peripheral endocannabinoid serum level in association with repetitive transcranial magnetic stimulation (rTMS) treatment in patients with major depressive disorder. Scientific Reports, 11(1), 8867. 10.1038/s41598-021-87840-5 33893327 PMC8065048

[brb33323-bib-0082] Mato, S. , Pilar‐Cuéllar, F. , Valdizán, E. M. , González‐Maeso, J. , Rodríguez‐Puertas, R. , Meana, J. , Sallés, J. , Crespo‐Facorro, B. , & Pazos, Á. (2018). Selective up‐regulation of cannabinoid CB1 receptor coupling to Go‐proteins in suicide victims with mood disorders. Biochemical Pharmacology, 157, 258–265. 10.1016/j.bcp.2018.08.012 30099006 PMC6263149

[brb33323-bib-0058] Molina, E. , Cervilla, J. , Rivera, M. , Torres, F. , Bellón, J. Á. , Moreno, B. , King, M. , Nazareth, I. , & Gutiérrez, B. (2011). Polymorphic variation at the serotonin 1‐A receptor gene is associated with comorbid depression and generalized anxiety. Psychiatric Genetics, 21(4), 195–201. 10.1097/YPG.0b013e3283457a48 21512427

[brb33323-bib-0059] Monteleone, P. , Bifulco, M. , Maina, G. , Tortorella, A. , Gazzerro, P. , Proto, M. C. , Di Filippo, C. , Monteleone, F. , Canestrelli, B. , & Buonerba, G. (2010). Investigation of CNR1 and FAAH endocannabinoid gene polymorphisms in bipolar disorder and major depression. Pharmacological Research, 61(5), 400–404. 10.1016/j.phrs.2010.01.002 20080186

[brb33323-bib-0060] Morena, M. , Patel, S. , Bains, J. S. , & Hill, M. N. (2016). Neurobiological interactions between stress and the endocannabinoid system. Neuropsychopharmacology: Official Publication of the American College of Neuropsychopharmacology, 41(1), 80–102. 10.1038/npp.2015.166 26068727 PMC4677118

[brb33323-bib-0061] Navarro, D. , Gasparyan, A. , Navarrete, F. , Torregrosa, A. B. , Rubio, G. , Marín‐Mayor, M. , Acosta, G. B. , Garcia‐Gutiérrez, M. S. , & Manzanares, J. (2022). Molecular alterations of the endocannabinoid system in psychiatric disorders. International Journal of Molecular Sciences, 23(9), 4764. 10.3390/ijms23094764 35563156 PMC9104141

[brb33323-bib-0062] Obermanns, J. , Flasbeck, V. , Steinmann, S. , Juckel, G. , & Emons, B. (2022). Investigation of the serotonergic activity and the serotonin content in serum and platelet, and the possible role of the serotonin transporter in patients with depression. Behavioral Sciences, 12(6), 178. 10.3390/bs12060178 35735388 PMC9220674

[brb33323-bib-0064] Pandey, D. K. , Mahesh, R. , Kumar, A. A. , Rao, V. S. , Arjun, M. , & Rajkumar, R. (2010). A novel 5‐HT(2A) receptor antagonist exhibits antidepressant‐like effects in a battery of rodent behavioural assays: Approaching early‐onset antidepressants. Pharmacology, Biochemistry, and Behavior, 94(3), 363–373. 10.1016/j.pbb.2009.09.018 19800913

[brb33323-bib-0065] Parsey, R. V. , Ogden, R. T. , Miller, J. M. , Tin, A. , Hesselgrave, N. , Goldstein, E. , Mikhno, A. , Milak, M. , Zanderigo, F. , Sullivan, G. M. , Oquendo, M. A. , & Mann, J. J (2010). Higher serotonin 1A binding in a second major depression cohort: Modeling and reference region considerations. Biological Psychiatry, 68(2), 170–178. 10.1016/j.biopsych.2010.03.023 20497898 PMC2900398

[brb33323-bib-0066] Parsey, R. V. , Olvet, D. M. , Oquendo, M. A. , Huang, Y.‐Y. , Ogden, R. T. , & Mann, J. J. (2006). Higher 5‐HT 1A receptor binding potential during a major depressive episode predicts poor treatment response: Preliminary data from a naturalistic study. Neuropsychopharmacology, 31(8), 1745–1749. 10.1038/sj.npp.1300992 16395308

[brb33323-bib-0067] Parsons, M. J. , D'souza, U. M. , Arranz, M.‐J. , Kerwin, R. W. , & Makoff, A. J. (2004). The–1438A/G polymorphism in the 5‐hydroxytryptamine type 2A receptor gene affects promoter activity. Biological Psychiatry, 56(6), 406–410. 10.1016/j.biopsych.2004.06.020 15364038

[brb33323-bib-0068] Pillai, R. L. I. , Bartlett, E. A. , Ananth, M. R. , Zhu, C. , Yang, J. , Hajcak, G. , Parsey, R. V. , & Delorenzo, C. (2020). Examining the underpinnings of loudness dependence of auditory evoked potentials with positron emission tomography. Neuroimage, 213, 116733. 10.1016/j.neuroimage.2020.116733 32169543 PMC7254571

[brb33323-bib-0069] Puig, M. V. (2005). Modulation of the activity of pyramidal neurons in rat prefrontal cortex by raphe stimulation in vivo: Involvement of serotonin and GABA. Cerebral Cortex (New York, N.Y.: 1991), 15(1), 1–14. 10.1093/cercor/bhh104 15238448

[brb33323-bib-0070] Rana, T. , Behl, T. , Sehgal, A. , Mehta, V. , Singh, S. , Kumar, R. , & Bungau, S. (2021). Integrating endocannabinoid signalling in depression. Journal of Molecular Neuroscience: MN, 71(10), 2022–2034. 10.1007/s12031-020-01774-7 33471311

[brb33323-bib-0071] Romero‐Sanchiz, P. , Nogueira‐Arjona, R. , Pastor, A. , Araos, P. , Serrano, A. , Boronat, A. , Garcia‐Marchena, N. , Mayoral, F. , Bordallo, A. , Alen, F. , Suárez, J. , De La Torre, R. , Pavón, F. J. , & Rodríguez De Fonseca, F. (2019). Plasma concentrations of oleoylethanolamide in a primary care sample of depressed patients are increased in those treated with selective serotonin reuptake inhibitor‐type antidepressants. Neuropharmacology, 149, 212–220. 10.1016/j.neuropharm.2019.02.026 30822499

[brb33323-bib-0072] Rouzer, C. A. , Ghebreselasie, K. , & Marnett, L. J. (2002). Chemical stability of 2‐arachidonylglycerol under biological conditions. Chemistry and Physics of Lipids, 119(1–2), 69–82. 10.1016/S0009-3084(02)00068-3 12270674

[brb33323-bib-0073] Schennach, R. , Zill, P. , Obermeier, M. , Hauer, D. , Dehning, S. , Cerovecki, A. , Opgen‐Rhein, M. , Musil, R. , Spellmann, I. , Matz, J. , Krause, D. , Seemüller, F. , Müller, N. , Möller, H.‐J. , Bondy, B. , & Riedel, M. (2012). The CNR1 gene in depression and schizophrenia‐is there an association with early improvement and response? Psychiatry Research, 196(1), 160. 10.1016/j.psychres.2011.11.021 22370152

[brb33323-bib-0074] Schreiber, D. , Harlfinger, S. , Nolden, B. M. , Gerth, C. W. , Jaehde, U. , Schömig, E. , Klosterkötter, J. , Giuffrida, A. , Astarita, G. , Piomelli, D. , & Markus Leweke, F. (2007). Determination of anandamide and other fatty acyl ethanolamides in human serum by electrospray tandem mass spectrometry. Analytical Biochemistry, 361(2), 162–168. 10.1016/j.ab.2006.11.027 17196922

[brb33323-bib-0075] Struik, D. , Sanna, F. , & Fattore, L. (2018). The modulating role of sex and anabolic‐androgenic steroid hormones in cannabinoid sensitivity. Frontiers in Behavioral Neuroscience, 12, 249. 10.3389/fnbeh.2018.00249 30416437 PMC6212868

[brb33323-bib-0076] Tabatadze, N. , Huang, G. , May, R. M. , Jain, A. , & Woolley, C. S. (2015). Sex differences in molecular signaling at inhibitory synapses in the hippocampus. Journal of Neuroscience, 35(32), 11252–11265. 10.1523/JNEUROSCI.1067-15.2015 26269634 PMC4532757

[brb33323-bib-0077] Tencomnao, T. , Thongrakard, V. , Phuchana, W. , Sritharathikhun, T. , & Suttirat, S. (2010). No relationship found between‐1438A/G polymorphism of the serotonin 2A receptor gene (rs6311) and major depression susceptibility in a northeastern Thai population. Genetics and Molecular Research, 9(2), 1171–1176. 10.4238/vol9-2gmr823 20589614

[brb33323-bib-0078] Wood, J. T. , Williams, J. S. , Pandarinathan, L. , Courville, A. , Keplinger, M. R. , Janero, D. R. , Vouros, P. , Makriyannis, A. , & Lammi‐Keefe, C. J. (2008). Comprehensive profiling of the human circulating endocannabinoid metabolome: Clinical sampling and sample storage parameters. Clinical Chemistry and Laboratory Medicine, 46(9), 1289–1295. 10.1515/CCLM.2008.242 18611105 PMC3733471

[brb33323-bib-0079] Yin, A.‐Q. , Wang, F. , & Zhang, X. (2019). Integrating endocannabinoid signaling in the regulation of anxiety and depression. Acta Pharmacologica Sinica, 40(3), 336–341. 10.1038/s41401-018-0051-5 30002489 PMC6460364

[brb33323-bib-0080] Zoerner, A. A. , Batkai, S. , Suchy, M.‐T. , Gutzki, F.‐M. , Engeli, S. , Jordan, J. , & Tsikas, D. (2012). Simultaneous UPLC–MS/MS quantification of the endocannabinoids 2‐arachidonoyl glycerol (2AG), 1‐arachidonoyl glycerol (1AG), and anandamide in human plasma: Minimization of matrix‐effects, 2AG/1AG isomerization and degradation by toluene solvent extraction. Journal of Chromatography B, 883–884, 161–171. 10.1016/j.jchromb.2011.06.025 21752730

